# Editorial: Artificial Membrane Transporters

**DOI:** 10.3389/fchem.2022.841159

**Published:** 2022-02-02

**Authors:** Changliang Ren, Pinaki Talukdar, Huaqiang Zeng

**Affiliations:** ^1^ School of Pharmaceutical Sciences, Xiamen University, Xiamen, China; ^2^ Shenzhen Research Institute of Xiamen University, Shenzhen, China; ^3^ Department of Chemistry, Indian Institute of Science Education and Research Pune, Pune, India; ^4^ School of Chemistry and Chemical Engineering, Northwestern Polytechnical University, Xi’an, China

**Keywords:** artificial membrane transporter, transmembrane transport, supramolecular chemistry, artificial ion channels, self-assembly, biomimetics

**Editorial on the Research Topic**

**Artificial Membrane Transporters**

Highly regulated exchanges of different molecular species (metabolites, water, ions, etc.) inside and outside living cells across cell membrane are of paramount significance to maintaining proper functions of biological systems. Membrane transport proteins (channels, carriers and pumps) precisely control the cross-membrane transport of ions and molecules as one way to coordinate cellular functions and physiological responses. Due to the structural complexity and often limited scalability of natural protein transporters, artificial transmembrane transporters are highly appealing as simplified models for understanding the mechanisms responsible for the material exchange between the intracellular and extracellular media, with promising applications as therapeutics for “channelopathies,” cancers and bacterial infections.

In this collection of five disparate research articles, we would like to highlight both experimental and computational efforts convergent toward creating artificial membrane transporters, with novel transmembrane transport properties, enhanced understanding of membrane transport mechanisms and therapeutic applications.

Maslowska-Jarzyna et al. explored a series of chloride transporters, which consist of 1,8-diamidocarbazoles decorated with highly electron withdrawing substituents. This research reveals that diamidocarbazoles with highly electron withdrawing nitro- and cyano-substituents in the carbazole core can transport chloride ion by forming a 1:1 complex with chloride and carrying it through lipid bilayers, which is particularly impressive considering their simple structure and easy availability. Surprisingly, 3,6-dinitro-substituted receptors gives a very low EC_50_ value of 22.4 nM in the chloride/nitrate exchange assay, placing this transporter as one of the most active artificial chloride transporters known to date. Intriguingly, it can also act as a pH-switchable transporter, with “ON” in acidic environment and “OFF” at neutral pH. The above-mentioned characteristics of 3,6-dinitro-substituted diamidocarbazole make this chloride transporter a promising lead compound for further biological studies.

Licsandru et al. underlines multivalent membrane-active K^+^- and H^+^-transporting species in relation to H-bonding-directed self-assembled superstructures from imidazole and 3-amino-triazole amphiphiles. The intriguing findings arising from this study reveal fluorinated transporters to be more active than the corresponding non-fluorinated counterparts and the R enantiomers to have a higher activity than the S enantiomers under the same conditions. Another interesting viewpoint raised by the authors is that “supramolecular polymorphism” can be associated with the formation of active structures within bilayer membranes.

Unlike most of the artificial membrane transporters that incorporate hydrophobic structures to facilitate molecular insertion into hydrophobic phospholipid bilayer membrane, McNally et al. (2008) reported an inspiring strategy that derivatizes a natural phosphatidylcholine lipid molecule with an anion-binding urea unit to achieve Cl^−^ transport via a relay mechanism. Applying the same strategy, Wang et al. modified the phospholipid molecule with a crown ether-based cation recognition group to generate phospholipid derivative **LC**. Very interestingly, **LC** facilitates efficient cation transport through a channel rather than a relay mechanism. Moreover, **LC** molecules readily disperse into water to form 20–30 nm vesicles, eliminating the use of organic solvents and the competitive self-precipitation in the physiological solution before inserting into lipid membrane. Besides, **LC** demonstrates obvious toxicity to HeLa cells in a dose-dependent manner. In combination with its good dispersity in water and bioavailability, **LC** holds the potential to become a nano-antitumor chemotherapeutic drug.

Peptide-appended pillar[5]arenes (PAP) with an angstrom-scale pore size (∼4.5 Å) are highly interesting and promising candidates for water transport owing to their conformational flexibility and high permeability. It is necessary to explore the water transport characteristics of this unique class of biomimetic pores in different membrane environments. Barden and Vashisth presents an inception-to-implementation description of the water dynamics triggered by PAP in biological (lipid) and biomimetic (block copolymer) membranes by using molecular dynamics simulations. Simulations uncover significant conformational flexibility in regions of the peptide arms away from the central rigid PAP ring and its adjacent regions, and show that insertion of PAP channels is more favorable in lipids with respect to the biomimetic membranes. Further, PAP channel preserves single channel permeabilities of low free energy barriers in each type of membrane, and the number of internal H-bonding sites can be nicely correlated to single file water diffusivity. These simulation results are in great accord with other synthetic and biological water channels having sub-nm pore sizes.

Self-assembling cyclic peptide nanotubes are appealing not only structurally for their cylinder shapes, tunable interior and exterior properties as well as expected biocompatibility but also functionally as potent transmembrane channel mimetics. A computational study on a new class of self-assembling peptide motifs containing alternatively arranged δ–aminocycloalkanecarboxylic acids and natural α–amino acids is reported by Blanco-González et al.. The structural and dynamical behavior of water and different cations and corresponding counter Cl^−^ anions inside this type of nanotubes are extensively studied by molecular dynamics simulation. Interestingly, water molecules are found to spatially distribute over the transversal plane, clearly defining a six-point star shape formed by a minimum of four water types (type I–IV). Unlike Li^+^ ions which are sequestered by the lipid phosphate groups and prevented from internalizing into the channel, Na^+^, K^+^, Cs^+^, and Ca^2+^ are found to internalize into the channel at different rates, proportions, and locations. Compared with cations, the presence of Cl^−^ anions inside the channels is relatively rare. Additionally, these ions residing in the channels show minimum effect on disturbing the exotic pattern created by filled water molecules. The simulation results provide new insights for a better understanding of the ions and water transport by cyclopeptides, allowing more rational strategies for improving channel activities.
